# Long-term efficacy, safety, and patient-reported outcomes of apitegromab in patients with spinal muscular atrophy: results from the 36-month TOPAZ study

**DOI:** 10.3389/fneur.2024.1419791

**Published:** 2024-07-22

**Authors:** Thomas O. Crawford, John W. Day, Darryl C. De Vivo, Jena M. Krueger, Eugenio Mercuri, Andres Nascimento, Amy Pasternak, Elena Stacy Mazzone, Tina Duong, Guochen Song, Jing L. Marantz, Scott Baver, Dongzi Yu, Lan Liu, Basil T. Darras

**Affiliations:** ^1^Johns Hopkins Medical, Baltimore, MD, United States; ^2^Stanford Neuroscience Health Center, Palo Alto, CA, United States; ^3^Departments of Neurology and Pediatrics, Columbia University Irving Medical Center, New York, NY, United States; ^4^Helen DeVos Children’s Hospital, Grand Rapids, MI, United States; ^5^Centro Clinico NeMO, Fondazione Policlinico Gemelli, IRCCS, Rome, Italy; ^6^Pediatric Neurology and Psychiatry, Catholic University, Rome, Italy; ^7^Neuromuscular Unit, Department of Neurology, Applied Research in Neuromuscular Diseases, Institut de Recerca Sant Joan de Déu (CIBERER), Barcelona, ISCIII, Esplugues de Llobregat, Spain; ^8^Department of Neurology, Boston Children’s Hospital, Harvard Medical School, Boston, MA, United States; ^9^Scholar Rock, Inc., Cambridge, MA, United States

**Keywords:** apitegromab, caregiver-reported outcomes, efficacy, long-term, motor function, safety, spinal muscular atrophy

## Abstract

**Background and purpose:**

At 12 months in the phase 2 TOPAZ study, treatment with apitegromab was associated with both an improved motor function in patients with Type 2 or 3 spinal muscular atrophy (SMA) and with a favorable safety profile. This manuscript reports the extended efficacy and safety in the nonambulatory group of the TOPAZ study at 36 months.

**Methods:**

Patients who completed the primary study (NCT03921528) could enroll in an open-label extension, during which patients received apitegromab 20 mg/kg by intravenous infusion every 4 weeks. Patients were assessed periodically via the Hammersmith Functional Motor Scale–Expanded (HFMSE), Revised Upper Limb Module (RULM), World Health Organization (WHO) motor development milestones, Pediatric Evaluation of Disability Inventory Computer Adaptive Test (PEDI-CAT) Daily Activities and Mobility domains, and Patient-Reported Outcomes Measurement Information System (PROMIS) Fatigue questionnaire.

**Results:**

Of the 58 patients enrolled in TOPAZ, 35 were nonambulatory (mean age 7.3 years). The mean change at 36 months in HFMSE score from baseline was +4.0 (standard deviation [SD]: 7.54), and + 2.4 (3.24) for RULM score (excluding *n* = 7 after scoliosis surgery). Caregiver-reported outcomes (PEDI-CAT and PROMIS Fatigue) showed improvements from baseline over 36 months. In addition, most patients (28/32) improved or maintained WHO motor milestones achieved at baseline. The most frequently reported treatment-emergent adverse events were pyrexia (48.6%), nasopharyngitis (45.7%), COVID-19 infection (40.0%), vomiting (40.0%), and upper respiratory tract infection (31.4%).

**Conclusion:**

The benefit of apitegromab treatment observed at 12 months was sustained at 36 months with no new safety findings.

## Introduction

Spinal muscular atrophy (SMA) is a neuromuscular disease characterized by the loss of motor neurons in the spinal cord and brain stem that results in progressive muscle weakness and atrophy of voluntary muscles of the limbs and trunk (1, 2). Impairment of motor function, mobility, and daily activities is also experienced individually, as is fatigue (3). SMA is caused by bi-allelic loss-of-function mutations in the survival motor neuron 1 (*SMN1*) gene on chromosome 5 (5q-SMA) (4). *SMN2*, an *SMN1* paralog, partially compensates for SMN protein deficiency. Variability in residual *SMN2* copy number between individuals with SMA is an important factor in the substantial clinical heterogeneity seen across SMA phenotypes (1). Although the classification is being altered by the introduction of SMN-directed treatments, SMA is traditionally divided into groups based upon the age at first appearance of weakness and the ultimate functional deficits and maximum level of motor function achieved. Individuals with early-onset, Type 1 SMA (age at onset: birth–6 months) are not expected to reach the milestone of unsupported sitting; for those with Type 2 SMA (onset <18 months), the expected maximal motor milestone is to sit independently, and for those with Type 3 SMA (onset >18 months), it is the ability to walk independently. Untreated, these achieved milestones are often lost over time (3). Approximately 80% of patients living with SMA have Type 2 or 3 (5).

Available therapies for SMA that enhance SMN protein expression have led to dramatic improvement in functional outcome inversely proportionate to the magnitude of motor neuron depletion at the time of treatment (6–8). They do not, however, affect the muscle atrophy and weakness associated with the partial denervation accumulated before treatment (2, 3). Consequently, substantial unmet need remains for a therapeutic strategy that increases function of the residual innervated muscle fibers that persist within partially denervated muscle. Apitegromab (SRK-015) is an investigational, fully human, immunoglobulin G4 monoclonal antibody that inhibits activation of myostatin, which is a transforming growth factor β superfamily–signaling molecule that negatively regulates skeletal muscle growth and strength (9). Myostatin is initially expressed in a latent, inactive form that is activated by subsequent enzymatic and non-enzymatic cleavage to release the active protein (1). By selectively binding to both pro- and latent forms of myostatin, apitegromab inhibits these activation steps, and thus the release of the mature myostatin growth factor (9). Inactivation of myostatin in animal models and myostatin mutations reported in children are associated with increased skeletal muscle mass and strength with no observed health consequences (10–12).

The efficacy and safety of apitegromab at 12 months in patients with Type 2 or 3 SMA in the phase 2 TOPAZ study were previously reported (13). Treatment with apitegromab was associated with a favorable safety profile and improved motor function outcomes as assessed by two ordinal scales created to assess motor functions impaired by SMA—the Hammersmith Functional Motor Scale–Expanded (HFMSE) and the Revised Upper Limb Module (RULM). Here we report longer-term efficacy and safety results from 36 months of treatment with apitegromab in nonambulatory patients with SMA enrolled in the TOPAZ open-label extension study.

## Materials and methods

### Study design and patients

TOPAZ (NCT03921528) is a multicenter, phase 2, active treatment study evaluating the efficacy and safety of apitegromab in patients with Type 2 or 3 SMA at 16 study sites in the United States and Europe. The study protocol and amendments were approved by the Independent Ethics Committee or Institutional Review Board and have been previously published (13). The study was designed and monitored in compliance with the ethical principles of the International Council for Harmonisation on Good Clinical Practice and in accordance with the Declaration of Helsinki. Before any screening visit activities were conducted, written informed consent was obtained from adult patients; for patients who were legally a minor, written informed consent was obtained from their parent/legal guardian and the patient’s written or oral assent was obtained, if applicable, in accordance with the regulatory and legal requirements of the participating location.

Methodological details for the study have been previously described (13). In brief, the study consisted of the 12-month primary treatment period previously reported and 3 successive 12-month extension periods, for a total of 48 months of treatment. Eligible patients were diagnosed with Type 2 or 3 SMA with 5q-SMA genotyping before receiving approved therapy for SMA.

Patients were enrolled into one of three cohorts. Cohort 1 consisted of individuals aged 5–21 years who were ambulatory, and thus classified as having Type 3 SMA; approximately half of this group was receiving nusinersen therapy. Cohort 2 comprised individuals aged 5–21 years with Type 2 or, now, nonambulatory Type 3 SMA who had all initiated nusinersen therapy after reaching 5 years of age. Cohort 3 consisted of nonambulatory individuals aged 2 years or older with Type 2 SMA who had all initiated their nusinersen therapy before 5 years of age. All patients received apitegromab by intravenous infusion every 4 weeks. During the primary treatment period, patients in Cohorts 1 and 2 received open-label apitegromab 20 mg/kg, while patients in Cohort 3 were double-blind randomized to a low 2-mg/kg, or a high 20-mg/kg apitegromab dose. In the extension periods, patients who received apitegromab 20 mg/kg in the primary treatment period continued their dose; patients originally receiving the low 2-mg/kg dose transitioned to the high 20-mg/kg apitegromab dose. The analyses presented here focus on the efficacy and safety in those study patients with nonambulatory SMA who received apitegromab for 36 months as part of the TOPAZ study.

### Efficacy outcomes

Motor function outcomes were assessed using the ordinal HFMSE (14, 15) and RULM (16) motor function scales created and validated for SMA studies. The RULM was used to assess patients aged ≥30 months at baseline (consistent with the RULM validation demographic). Investigators also assessed the attainment of six gross motor development milestones using World Health Organization (WHO) criteria: sitting without support, standing with assistance, hands-and-knees crawling, standing alone, walking with assistance, and walking alone (17).

Caregiver-reported outcomes on daily activities and mobility skills in a patient natural habitat were assessed using the Daily Activities and Mobility domains of the Pediatric Evaluation of Disability Inventory Computer Adaptive Test (PEDI-CAT) (18, 19). The PEDI-CAT was a caregiver-completed assessment. Fatigue was assessed using the Patient-Reported Outcomes Measurement Information System (PROMIS) Fatigue questionnaire (20). The PROMIS Fatigue questionnaire measures a range of symptoms from mild subjective feelings of tiredness to an overwhelming, debilitating, and sustained sense of exhaustion, with higher scores reflecting more fatigue. In this study, the PROMIS Fatigue questionnaire utilized a caregiver proxy for completion, as well as a patient self-report if the patient was ≥8 years of age. For uniformity, all data presented here were from a caregiver proxy.

### Safety assessments

Safety assessments included the reporting of treatment-emergent adverse events (TEAEs), physical examinations, vital signs, clinical laboratory testing, 12-lead electrocardiograms, and anti-drug antibody testing.

### Statistical analyses

Mean changes from baseline at 6, 12, 24, and 36 months were summarized for HFMSE total scores, RULM total scores, PEDI-CAT Daily Activities and Mobility domain scaled scores, and PROMIS Fatigue scores. An observed case analysis was used that included data from all nonambulatory patients (Cohorts 2 and 3) and was based on the available data for given timepoints. The analysis population included patients in Cohorts 2 and 3 receiving high-dose (20 mg/kg) apitegromab and patients in Cohort 3 receiving low-dose (2 mg/kg) apitegromab who transitioned to 20 mg/kg in the extension period. Motor function was also assessed separately in the subgroup of patients aged 2–12 years. Data that were obtained after scoliosis surgery were excluded for HFMSE and RULM because such surgery was known to be associated with a decline in motor milestone scores in the months following the surgery, thus confounding motor function assessment (21). Mean changes are presented with corresponding standard deviation (SD). Data on attainment of WHO gross motor development milestones are reported descriptively.

## Results

### Patients

Of 58 patients enrolled in the TOPAZ study, 23 ambulatory patients were allocated to Cohort 1, and 15 and 20 nonambulatory patients were allocated to Cohorts 2 and 3, respectively. Fifty-seven patients completed the primary treatment period and enrolled in the extension study; of these, 35 were nonambulatory and were the focus of the efficacy and safety data presented here. A study flow diagram is shown in Supplementary Figure S1.

Baseline patient characteristics for the TOPAZ nonambulatory population (Cohorts 2 and 3) are shown in Table 1. The mean age at baseline for the total nonambulatory population was 7.3 years (range: 2–19 years). Overall, 25 patients (71.4%) had a history of contractures, and 18 patients (51.4%) had a history of scoliosis. Most patients (77.1%) had three copies of *SMN2*. In the subgroup of 2–12-year-olds, the mean age was 5.5 years (range: 2–11 years; Supplementary Table 1).

**Table 1:** Baseline patient characteristics by cohort.

	Cohort 2 (*n* = 15)	Cohort 3 (*n* = 20)	Total nonambulatory population (*N* = 35)
Age at informed consent, years, mean (min, max)	11.7 (8, 19)	4.0 (2, 6)	7.3 (2, 19)
Age at symptom onset, years, mean (min, max)	1.35 (0.5, 2.0)	0.95 (0.5, 3.5)	1.12 (0.5, 3.5)
Sex, *n* (%)			
Female	8 (53.3)	8 (40.0)	16 (45.7)
Male	7 (46.7)	12 (60.0)	19 (54.3)
Race, *n* (%)			
Asian	2 (13.3)	1 (5.0)	3 (8.6)
Black or African American	1 (6.7)	1 (5.0)	2 (5.7)
White or Other	12 (80.0)	18 (90.0)	30 (85.7)
SMA history, *n* (%)			
Contractures	13 (86.7)	12 (60.0)	25 (71.4)
Scoliosis	11 (73.3)	7 (35.0)	18 (51.4)
SMN therapy duration, months, mean (min, max)	24.2 (11.8, 39.3)	24.0 (9.7, 34.2)	24.1 (9.7, 39.3)
*SMN2* copy number, *n* (%)			
2	0 (0.0)	2 (10.0)	2 (5.7)
3	11 (73.3)	16 (80.0)	27 (77.1)
4	2 (13.3)	1 (5.0)	3 (8.6)
No response	2 (13.3)	1 (5.0)	3 (8.6)
HFMSE score, mean (min, max)	22.7 (13, 39)	24.8 (12, 44)	23.9 (12, 44)
RULM score, mean (min, max)	26.6 (19, 34)	23.8 (15, 34)^a^	25.1 (15, 34)

a Data are for *n* = 19 patients.

HFMSE, Hammersmith Functional Motor Scale–Expanded; max, maximum; min, minimum; RULM, Revised Upper Limb Module; SMA, spinal muscular atrophy; SMN, survival motor neuron.

### Efficacy outcomes

**Figure 1:**
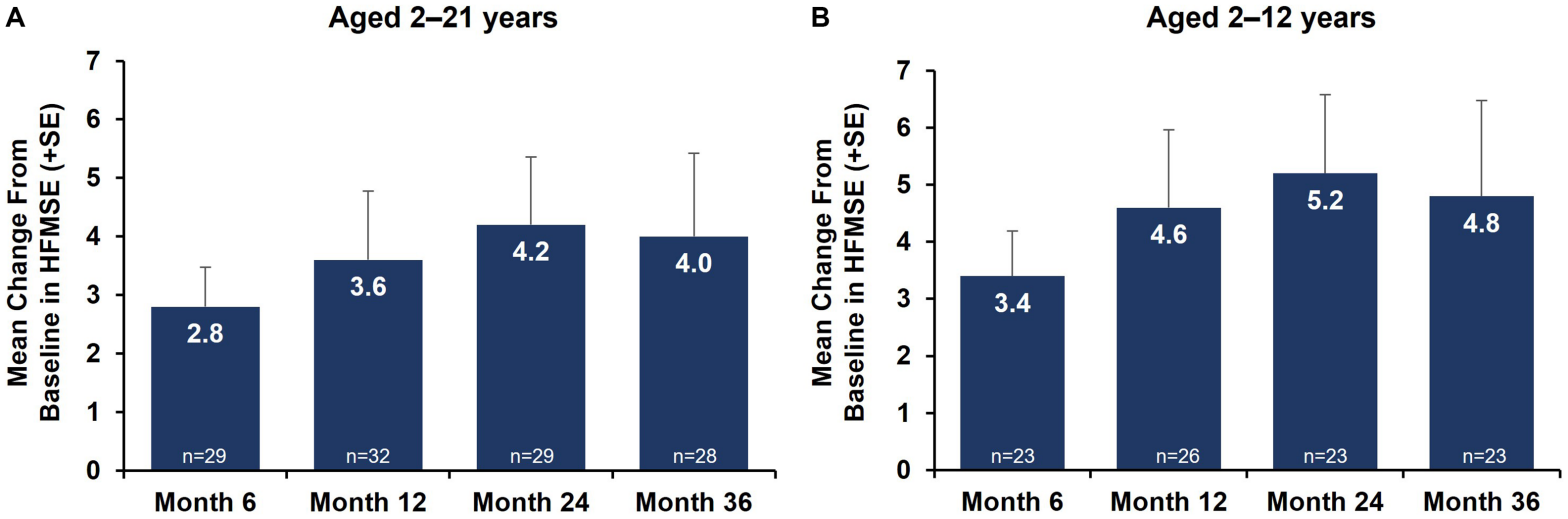
Mean change from baseline in motor function outcomes by HFMSE over 36 months [nonambulatory SMA, aged 2–21 years **(A)** and aged 2–12 years **(B)**]. Error bars represent standard error of means. This analysis population included patients receiving either low-dose (2 mg/kg) or high-dose (20 mg/kg) apitegromab (inclusive of patients in Cohort 3 who transitioned from 2 mg/kg to 20 mg/kg in Year 2). This analysis excludes data post scoliosis surgery from 7 patients. HFMSE, Hammersmith Functional Motor Scale–Expanded; SE, standard error; SMA, spinal muscular atrophy.

**Figure 2:**
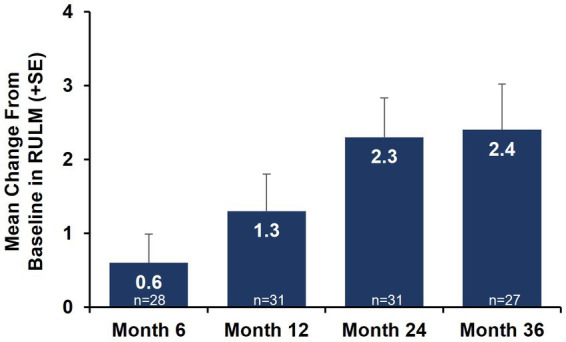
Mean change from baseline in motor function outcomes by RULM (nonambulatory SMA, aged 2–21 years). Error bars represent standard error of means. This analysis population included patients receiving either low-dose (2 mg/kg) or high-dose (20 mg/kg) apitegromab (inclusive of patients in Cohort 3 who transitioned from 2 mg/kg to 20 mg/kg in Year 2). This analysis excludes data post scoliosis surgery from 6 patients. RULM, Revised Upper Limb Module; SE, standard error; SMA, spinal muscular atrophy.

Motor function outcomes as assessed by HFMSE showed sustained improvements from baseline over 36 months in both the total nonambulatory population ages 2–21 years and the subgroup aged 2–12 years (Figure 1). Mean change (SD) from baseline in HFMSE total score at 36 months was +4.0 (7.54) in nonambulatory patients overall and + 4.8 (8.05) in the subgroup aged 2–12 years. Upper limb function as assessed by RULM showed sustained improvements from baseline over 36 months (Figure 2; Supplementary Figure S2). Mean change (SD) from baseline in RULM total score at 36 months was +2.4 (3.24) in nonambulatory patients overall and + 2.8 (3.20) in the subgroup aged 2–12 years. Both HFMSE and RULM analyses exclude data from seven patients due to scoliosis surgery.

Over 36 months, 88% (28/32) of patients with assessments at 36 months had improved or maintained WHO motor milestones that they had achieved at baseline. Excluding those who had scoliosis surgery, 93% (25/27) of patients improved or maintained baseline WHO motor milestones. Of 20 patients receiving nusinersen earlier than 5 years of age, 6 gained new WHO motor milestones, including 2 who were able to walk independently.

**Figure 3:**
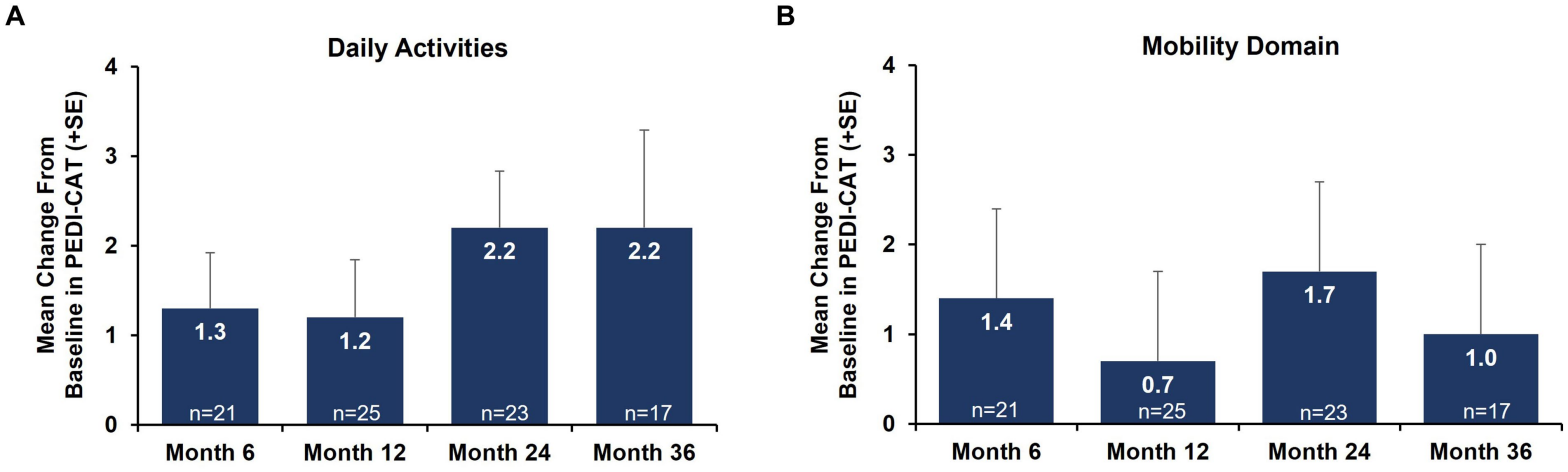
Mean change from baseline in PEDI-CAT daily activities **(A)** and mobility domain **(B)** nonambulatory SMA, aged 2–21 years. Error bars represent standard error of means. This analysis population included patients receiving either low-dose (2 mg/kg) or high-dose (20 mg/kg) apitegromab (inclusive of patients in Cohort 3 who transitioned from 2 mg/kg to 20 mg/kg in Year 2). PEDI-CAT, Pediatric Evaluation of Disability Inventory Computer Adaptive Test; SE, standard error; SMA, spinal muscular atrophy.

**Figure 4:**
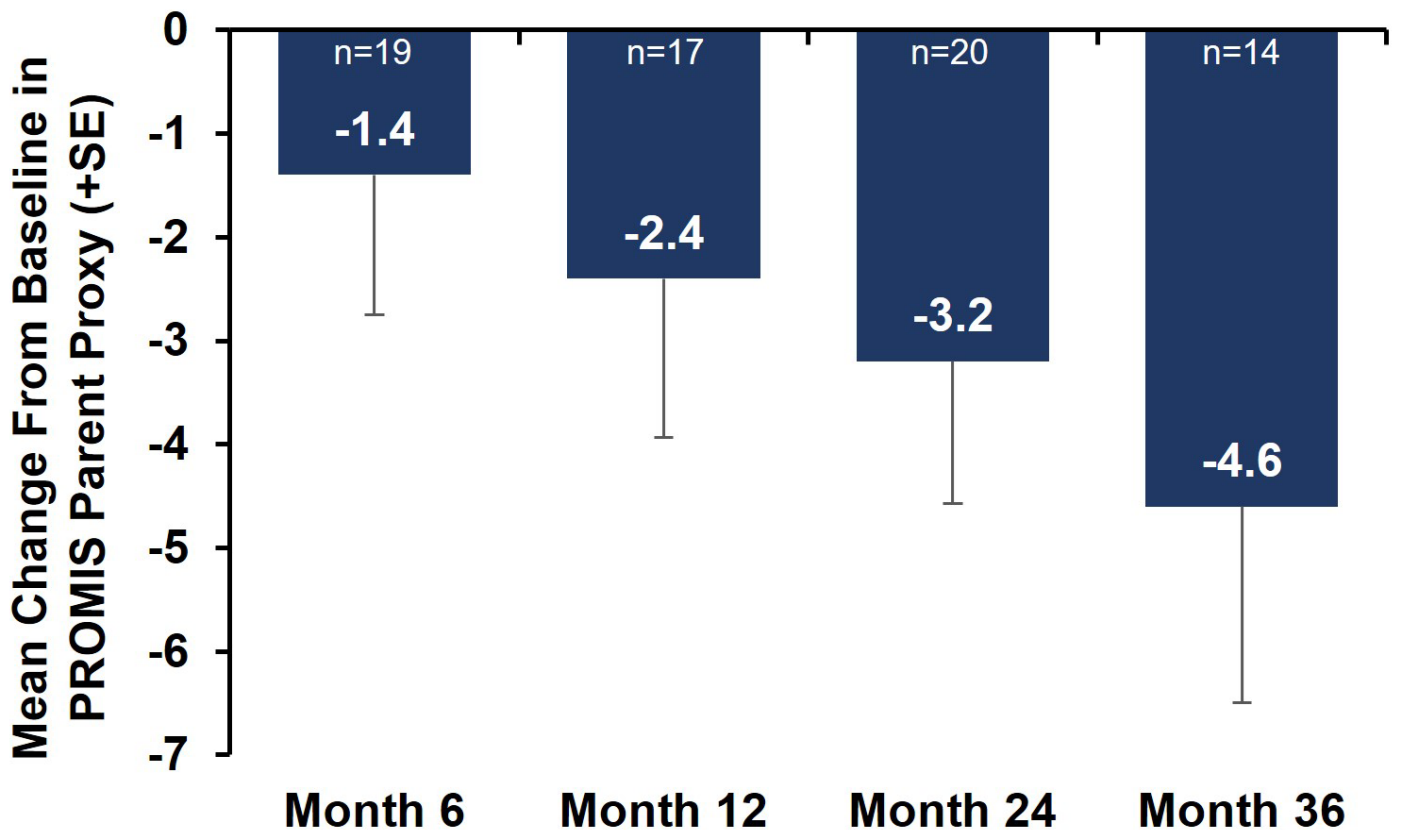
Mean change from baseline in PROMIS Fatigue questionnaire via caregiver proxy (nonambulatory SMA, aged 2–21 years). Error bars represent the standard error of means. This analysis population included nonambulatory patients from Cohorts 2 and 3, 2–21 years old receiving either low-dose (2 mg/kg) or high-dose (20 mg/kg) apitegromab (inclusive of patients in Cohort 3 who transitioned from 2 mg/kg to 20 mg/kg in Year 2). PROMIS, Patient-Reported Outcomes Measurement Information System; SE, standard error; SMA, spinal muscular atrophy.

**Figure 5:**
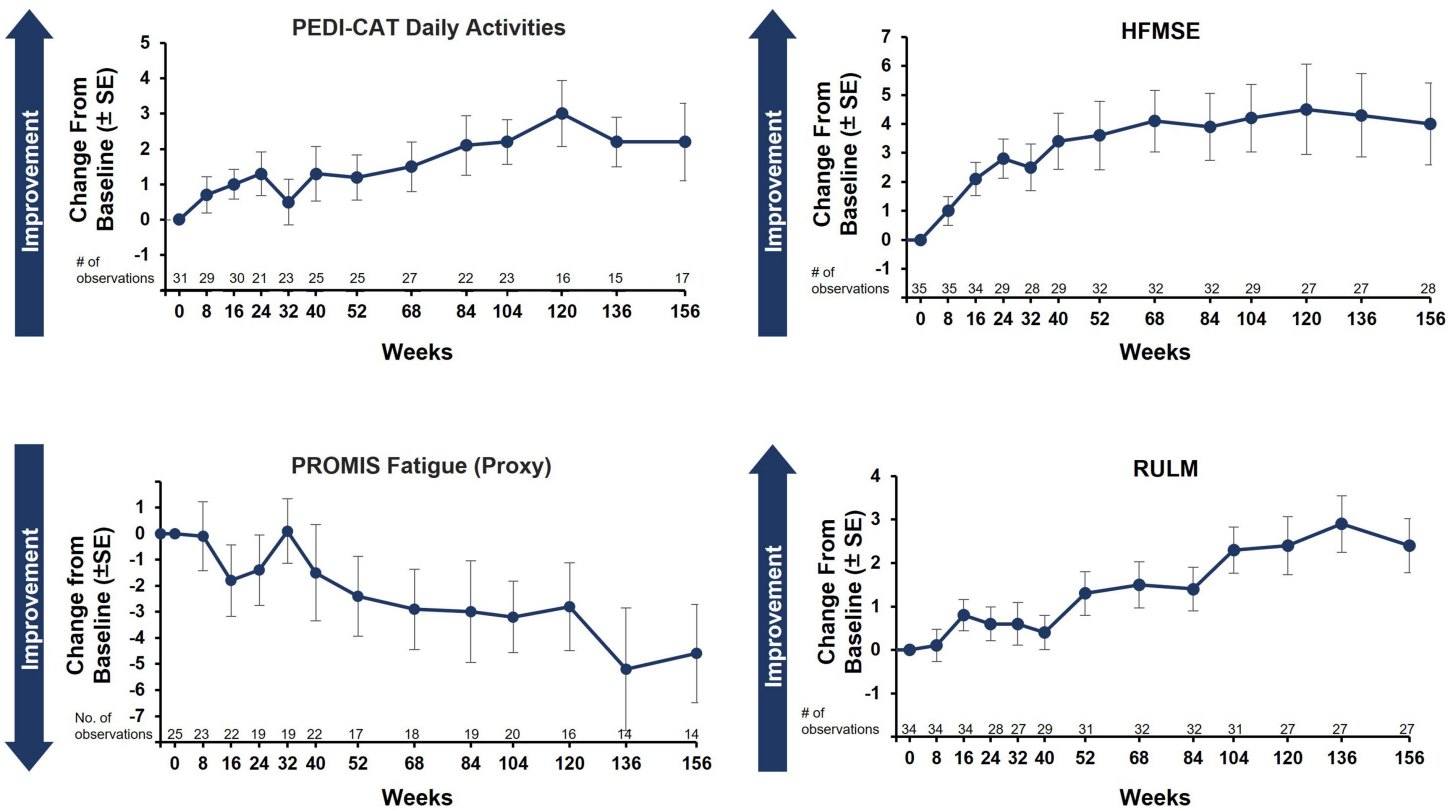
Caregiver-reported outcomes and motor function measures over 36 months (nonambulatory SMA, aged 2–21 years). Of note: Correlation tests were not performed. Post scoliosis surgery data were excluded for HFMSE and RULM. HFMSE, Hammersmith Functional Motor Scale–Expanded; PEDI-CAT, Pediatric Evaluation of Disability Inventory Computer Adaptive Test; PROMIS, Patient-Reported Outcomes Measurement Information System; RULM, Revised Upper Limb Module; SE, standard error; SMA, spinal muscular atrophy.

Caregiver-reported outcomes on daily activities as assessed by the caregiver-completed PEDI-CAT showed sustained improvement in daily activities over 36 months, with a change from baseline of 2.2 at Month 36 (Figure 3A). A positive change from baseline was also observed over 36 months in mobility domain scores, with a change from baseline of 1.0 observed at Month 36 (Figure 3B). Symptoms of fatigue as assessed by the PROMIS Fatigue questionnaire via caregiver proxy showed sustained improvements over the 36-month period, with the change from baseline at Month 36 being −4.6 compared to −2.4 at Month 12 (Figure 4). The changes over time in the PEDI-CAT and PROMIS Fatigue questionnaire caregiver-reported outcomes were consistent with changes over time in motor function measures (HFMSE and RULM; Figure 5).

### Safety

The safety profile observed for the nonambulatory patients was consistent with that of the overall study population (13). Of nonambulatory patients who received apitegromab for 36 months, 35 patients (100%) experienced at least one TEAE (Table 2; Supplementary Table 2). All drug-related TEAEs were mild to moderate in severity. There was no reported serious study drug–related TEAEs. The most frequently reported TEAEs were pyrexia (48.6%), nasopharyngitis (45.7%), COVID-19 infection (40.0%), vomiting (40.0%), and upper respiratory tract infection (31.4%). Fourteen patients (40.0%) experienced at least one serious TEAE. No serious TEAEs were considered related to the study drug. No deaths or suspected unexpected serious adverse reactions or hypersensitivity reactions to apitegromab were reported and no patients displayed positive titers for apitegromab antibodies.

**Table 2:** Safety summary over 36 months.

	Apitegromab 2 mg/kg* (*n* = 10)	Apitegromab 20 mg/kg (*n* = 25)	Total nonambulatory population (*N* = 35)
Any TEAE**, *n* (%)	10 (100.0)	25 (100.0)	35 (100.0)
Any serious TEAE, n (%)	5 (50.0)	9 (36.0)	14 (40.0)
Any TEAE leading to study drug discontinuation, *n* (%)	0 (0.0)	0 (0.0)	0 (0.0)
Any TEAE leading to death, *n* (%)	0 (0.0)	0 (0.0)	0 (0.0)
TEAEs in ≥ 25% of the total nonambulatory population, *n* (%)			
Pyrexia	4 (40.0)	13 (52.0)	17 (48.6)
Nasopharyngitis	5 (50.0)	11 (44.0)	16 (45.7)
COVID-19	6 (60.0)	8 (32.0)	14 (40.0)
Vomiting	5 (50.0)	9 (36.0)	14 (40.0)
Upper respiratory tract infection	3 (30.0)	8 (32.0)	11 (31.4)
Cough	4 (40.0)	7 (28.0)	11 (31.4)
Nausea	5 (50.0)	6 (24.0)	11 (31.4)
Headache	4 (40.0)	7 (28.0)	11 (31.4)
Scoliosis	1 (10.0)	9 (36.0)	10 (28.6)
Rash	2 (20.0)	7 (28.0)	9 (25.7)

*Transitioned to 20 mg/kg in Year 2. **Defined as adverse events that start after the first dose of study drug or start prior to the administration of study drug and worsen in severity/grade or relationship to investigational medication after the administration of study drug. COVID-19, Coronavirus disease 2019; TEAE, treatment-emergent adverse event.

## Discussion

We previously reported the substantial functional gains observed in individuals with Type 2 or 3 SMA receiving apitegromab in the primary 12-month treatment period of the TOPAZ study (13). This analysis of the long-term efficacy in the extension phase of TOPAZ, as assessed by ordinal outcome measures, HFMSE and RULM, and patient-reported measures, showed maintenance and continued improvement of meaningful motor function gains at 36 months.

Greater improvement in motor function was also observed in the subgroup of patients aged 2–12 years that defines the population being evaluated in the ongoing pivotal phase 3 SAPPHIRE study (NCT05156320). Importantly, of the 20 Cohort 3 patients who had first received SMN-expression–enhancing nusinersen therapy at an age earlier than 5 years, 6 gained new WHO motor milestones, including 2 who were able to walk independently. Although all patients in this open-label study received nusinersen, this improvement stands above reported outcomes in the steady maintenance phase of nusinersen treatment when observed gains generally plateau (22).

Understanding an individual’s everyday experience of function (e.g., home or school setting) is important to contextualizing the impact of therapies for SMA. The PEDI-CAT Daily Activities and Mobility domains are valid, discriminative, and feasible performance-based caregiver-reported outcome measures in SMA (23). Both PEDI-CAT domains improved over 36 months. The daily activities domain predominantly assesses upper limb function (e.g., getting dressed, keeping clean, eating/mealtime, home tasks). Consistent with improvements in RULM, improvements on PEDI-CAT daily activities domains were maintained over 36 months with apitegromab treatment. In this nonambulatory population, despite the mobility domain primarily assessing ambulation (standing, walking, running), it is noteworthy that the scaled scores remained improved from baseline over 36 months.

In addition to impairments in motor function and muscle strength, perceived fatigue is a commonly reported symptom in patients living with SMA (24) and is a critical domain contributing to health-related quality of life (25). In a prospective longitudinal monocentric observational cohort study in adults receiving nusinersen, 75% of participants reported abnormal levels of fatigue at baseline, and before SMN-enhancing therapy, 53% stated that fatigue was one of their most disabling symptoms (26). At the time of TOPAZ study initiation, the PROMIS Fatigue measure was selected to assess the severity and impact of physical and cognitive fatigue. Sustained improvement from baseline over 36 months was observed in the caregiver proxy PROMIS Fatigue questionnaire. Studies with a larger patient population are warranted to further correlate caregiver-reported outcomes with functional outcomes.

The introduction of three SMN-expression–enhancing therapies (nusinersen, risdiplam, and onasemnogene abeparvovec) has remarkably changed the course of SMA in recent years. The effect of these therapies is related to the age at time of treatment and the degree of ongoing degeneration prior to therapy initiation; yet to be known is any potential longer-term limitation of treatment durability. However, for the present and foreseeable future, there remains substantial unmet medical need related to existing denervation muscle atrophy (3, 27). A therapy targeting an increase of muscle strength of remaining innervated muscle fibers is ideally suited to address this need, restoring motor function, fatigue, and overall quality of life (28). CURE SMA recently published the results of two large-scale surveys, assessing disease characteristics, healthcare, and burden of disease from individuals with SMA and their caregivers. In this real-world study, 50–81% of individuals with SMA had received at least 1 dose of nusinersen, yet less than half of those with SMA Type 2 or 3 were able to sit unsupported (SMA Type 2) or walk independently (SMA Type 3) (29). Despite 1 year of nusinersen therapy in a prospective longitudinal cohort study, general fatigue and physical fatigue tended to decrease, while mental fatigue and reduced motivation increased during treatment (26). Moreover, after 26 months of treatment with nusinersen in patients aged 6 months or older, despite a significant increase in HFMSE scores, PEDI-CAT mobility and daily activities domain scores did not significantly change over time (30). Conversely in the TOPAZ study, PEDI-CAT and PROMIS Fatigue questionnaire caregiver-reported outcomes improved over the course of 36 months; these changes were consistent with changes observed over time in motor function measures (HFMSE and RULM).

No new safety findings were identified with 36 months of apitegromab treatment, reinforcing a favorable longer-term safety profile. TEAEs were mild to moderate in severity and generally consistent with the underlying SMA patient population. The favorable safety profile of apitegromab is consistent with the specific and selective nature of its mechanism of action. The upstream targeting of the pro- and latent forms of myostatin in the skeletal muscle and the highly selective nature of the action has the potential to avoid undesirable off-target effects.

A key limitation of the current study is that the TOPAZ extension did not include a placebo or nusinersen monotherapy as a comparator. Also, additional variables may account for improvements in motor function, such as improvements from development or contribution of treatment with nusinersen. Patients enrolled in the TOPAZ study had received nusinersen for a mean of approximately 2 years, well into the steady maintenance phase of the nusinersen treatment, thus making it easier to isolate the effect of apitegromab. The lack of a clear correlation between HFMSE score and duration of prior nusinersen exposure also suggests that the improvement in motor function in the TOPAZ study is likely attributed to apitegromab. An additional point to consider is the clear dose relationship seen in the primary analysis of the TOPAZ results, with greater improvement in motor function seen with the 20-mg/kg apitegromab group than with the 2-mg/kg apitegromab group (13).

Results of the TOPAZ study at 36 months reinforce the longer-term benefit of apitegromab treatment in patients with SMA, with continued and sustained improvement across both clinical and caregiver-reported outcomes as well as a longer-term favorable safety profile. Moreover, the data highlight the therapeutic potential of apitegromab to address persistent weakness and fatigue in individuals with nonambulatory SMA, enabling patients to potentially improve their motor function and be able to maintain these improvements. These findings support further development of apitegromab in nonambulatory SMA. The ongoing phase 3 SAPPHIRE trial will further assess the efficacy and safety of apitegromab in individuals with Type 2 and nonambulatory Type 3 SMA receiving SMN-dependent therapy (either nusinersen or risdiplam) (31).

## Data availability statement

The original contributions presented in the study are included in the article/Supplementary material, further inquiries can be directed to the corresponding author.

## Ethics statement

The studies involving humans were approved by IEC/IRB Name/Address “Boston Children’s Hospital Office of Clinical Investigations 300 Longwood Ave. Boston, MA 02115 United States” “Oregon Health & Science University (OHSU) 3,181 SE Sam Jackson Park Rd., Mail Code L106-R1 Portland, OR United States” “Columbia University Medical Center IRB 154 Haven Avenue, First Floor New York, NY 10032 United States” “Advarra 6,940 Columbia Gateway Dr., Suite 110 Columbia, MD 21046 United States” “Stanford University Human Subjects Review Board 3,000 El Camino Real Palo Alto, CA 94305 United States” “Western Institutional Review Board (WIRB) 1,019 39th Avenue SE, Suite 120 Puyallup, WA 98374 United States” “The Johns Hopkins Medicine IRBs 1,620 McElderry Street, Reed Hall Baltimore, MD 21205 United States” “Advarra 6,940 Columbia Gateway Dr.; Suite 110 Columbia, MD 21046 United States” “Advarra 6,940 Columbia Gateway Dr., Suite 110 Columbia, MD 21046 United States” “Phoenix Children’s Hospital 1919 E. Thomas Rd. Phoenix, AZ 85016 United States” IEC/IRB Name/Address “Spectrum Health Human Research Protection Program 15 Michigan Street NE, Suite 701 Grand Rapids, MI 49503 United States” “Comitato Etico della Fondazione Policlinico Universitario Agostino Gemelli IRCCS Università Cattolica del Sacro Cuore IRCCS Fondazione Policlinico Universitario A. Gemelli Largo Agostino Gemelli 8 Roma, Italy 00168” “Comitato Etico Milano AREA 3 Piazza Ospedale Maggiore 3 Milano, Italy 20,162” “CEIC Fundacio Sant Joan de Deu Editha Tacbas Esplugues de Llobregat, Spain 08950” “UMC Utrecht T.a.v METC Huispost D.01.343, Postbus 85,500 Utrecht, Netherlands 3,508 GA.” The studies were conducted in accordance with the local legislation and institutional requirements. Written informed consent for participation in this study was provided by the participants’ legal guardians/next of kin.

## Author contributions

TC: Writing – original draft, Writing – review & editing. JD: Writing – original draft, Writing – review & editing. DV: Writing – original draft, Writing – review & editing. JK: Writing – original draft, Writing – review & editing. EM: Writing – original draft, Writing – review & editing. AN: Writing – original draft, Writing – review & editing. AP: Writing – original draft, Writing – review & editing. ESM: Writing – original draft, Writing – review & editing. TD: Writing – original draft, Writing – review & editing. GS: Writing – original draft, Writing – review & editing. JM: Writing – original draft, Writing – review & editing. SB: Writing – original draft, Writing – review & editing. DY: Writing – original draft, Writing – review & editing. LL: Writing – original draft, Writing – review & editing. BD: Writing – original draft, Writing – review & editing.
