# Supplementary material for: Long-term efficacy, safety, and patient-reported outcomes of apitegromab in patients with spinal muscular atrophy: results from the 36-month TOPAZ study
Source: Front Neurol. 2024 Jul 22;15:1419791. doi: 10.3389/fneur.2024.1419791 (PMC11299114; doi:10.3389/fneur.2024.1419791)
Supplement: Supplementary file 1 [file Data_Sheet_1.pdf]

SUPPLEMENTARY MATERIAL

SUPPLEMENTARY TABLE 1 Baseline patient characteristics by cohort (nonambulatory population aged 2–12).

	**Cohort 2** (*n* = 9)	**Cohort 3** (*n* = 20)	**Total Nonambulatory Population aged 2–12 years** (*N* = 29)
**Age at baseline, years, mean (min, max)**	8.9 (8.0, 11.0)	4.0 (2.0, 6.0)	5.5 (2.0, 11.0)
**Age at symptom onset, years, mean (min, max)**	1.31 (0.5, 2.0)	0.95 (0.5, 3.5)	1.06 (0.5, 3.5)
**Sex, *n* (%)**			
Female	6 (66.7)	8 (40.0)	14 (48.2)
Male	3 (33.3)	12 (60.0)	15 (51.7)
**Race, *n* (%)**			
Asian	1 (11.1)	1 (5.0)	2 (6.9)
Black or African American	0 (0.0)	1 (5.0)	1 (3.4)
White or Other	8 (88.9)	18 (90.0)	26 (89.7)
**SMA history, n (%)**			
Contractures	8 (88.9)	12 (60.0)	20 (69.0)
Scoliosis	7 (77.8)	7 (35.0)	14 (48.3)
**SMN therapy duration, months, mean (min, max)**	25.94 (16.5, 39.3)	24.02 (9.7, 34.2)	24.62 (9.7, 39.3)
***SMN2* copy number, *n* (%)**			
2	0 (0.0)	2 (10.0)	2 (6.9)
3	8 (88.9)	16 (80.0)	24 (82.8)
4	0 (0.0)	1 (5.0)	1 (3.4)
No response	1 (11.1)	1 (5.0)	2 (6.9)
**HFMSE score, mean (min, max)**	21.8 (13, 39)	24.8 (12, 44)	23.9 (12, 44)
**RULM score, mean (min, max)**	25.9 (19, 34)	23.8 (15, 34)	24.5 (15, 34)

HFMSE, Hammersmith Functional Motor Scale–Expanded; max, maximum; min, minimum; RULM, Revised Upper Limb Module; SMA, spinal muscular atrophy; SMN, survival motor neuron.

SUPPLEMENTAL TABLE 2 Treatment-emergent adverse events* occurring in ≥10% of the total nonambulatory patient population.

	Apitegromab 2 mg/kg** (*n* = 10)	Apitegromab 20 mg/kg (*n* = 25)	Total Nonambulatory Population (*N* = 35)
Pyrexia, *n* (%)	4 (40.0)	13 (52.0)	17 (48.6)
Nasopharyngitis, *n* (%)	5 (50.0)	11 (44.0)	16 (45.7)
COVID-19, *n* (%)	6 (60.0)	8 (32.0)	14 (40.0)
Vomiting, *n* (%)	5 (50.0)	9 (36.0)	14 (40.0)
Upper respiratory tract infection, *n* (%)	3 (30.0)	8 (32.0)	11 (31.4)
Cough, *n* (%)	4 (40.0)	7 (28.0)	11 (31.4)
Nausea, *n* (%)	5 (50.0)	6 (24.0)	11 (31.4)
Headache, *n* (%)	4 (40.0)	7 (28.0)	11 (31.4)
Scoliosis, *n* (%)	1 (10.0)	9 (36.0)	10 (28.6)
Rash, *n* (%)	2 (20.0)	7 (28.0)	9 (25.7)
Muscle contracture, *n* (%)	2 (20.0)	5 (20.0)	7 (20.0)
Nasal congestion, *n* (%)	2 (20.0)	5 (20.0)	7 (20.0)
Oropharyngeal pain, *n* (%)	1 (10.0)	6 (24.0)	7 (20.0)
Joint dislocation, *n* (%)	4 (40.0)	3 (12.0)	7 (20.0)
Back pain, *n* (%)	1 (10.0)	5 (20.0)	6 (17.1)
Joint contracture, *n* (%)	2 (20.0)	4 (16.0)	6 (17.1)
Rhinorrhea, *n* (%)	3 (30.0)	3 (12.0)	6 (17.1)
Abdominal pain upper, *n* (%)	2 (20.0)	4 (16.0)	6 (17.1)
Constipation, *n* (%)	2 (20.0)	4 (16.0)	6 (17.1)
Diarrhea, *n* (%)	2 (20.0)	4 (16.0)	6 (17.1)
Scoliosis surgery, *n* (%)	0 (0.0)	6 (24.0)	6 (17.1)
Ear infection, *n* (%)	2 (20.0)	3 (12.0)	5 (14.3)
Influenza, *n* (%)	2 (20.0)	3 (12.0)	5 (14.3)
Arthralgia, *n* (%)	0 (0.0)	5 (20.0)	5 (14.3)
Tachycardia, *n* (%)	1 (10.0)	4 (16.0)	5 (14.3)
Eczema, *n* (%)	2 (20.0)	2 (8.0)	4 (11.4)
Erythema, *n* (%)	0 (0.0)	4 (16.0)	4 (11.4)
Fall, *n* (%)	2 (20.0)	2 (8.0)	4 (11.4)
Tibia fracture, *n* (%)	0 (0.0)	4 (16.0)	4 (11.4)
Vitamin D deficiency, *n* (%)	0 (0.0)	4 (16.0)	4 (11.4)
Hypotension, *n* (%)	1 (10.0)	3 (12.0)	4 (11.4)

*Defined as adverse events that start after the first dose of study drug or start prior to the administration of study drug and worsen in severity/grade or relationship to investigational medication after the administration of study drug.
**Transitioned to 20 mg/kg in Year 2. COVID-19, Coronavirus disease 2019.

SUPPLEMENTARY FIGURE S1 Study flow diagram.

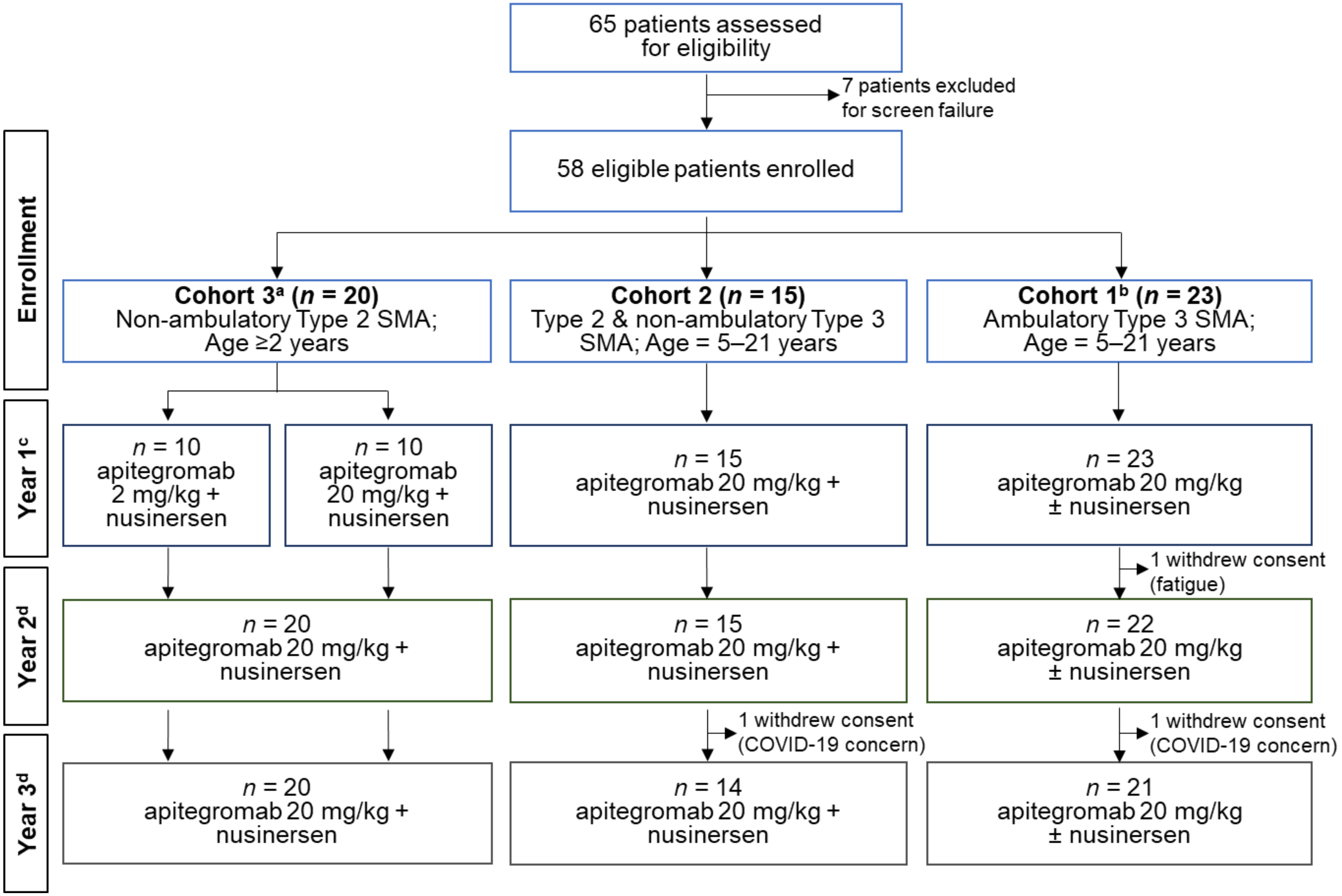

[a]Patients randomized to receive 2- or 20-mg/kg apitegromab. [b]Patients stratified based on previous treatment with approved SMN therapy. [c]Primary study period. [d]Study extension period. COVID-19, Coronavirus disease 2019; SMA, spinal muscular atrophy; SMN, survival motor neuron.

SUPPLEMENTARY FIGURE S2 Mean change from baseline in motor function outcomes by RULM (nonambulatory SMA, aged 2–12 years).

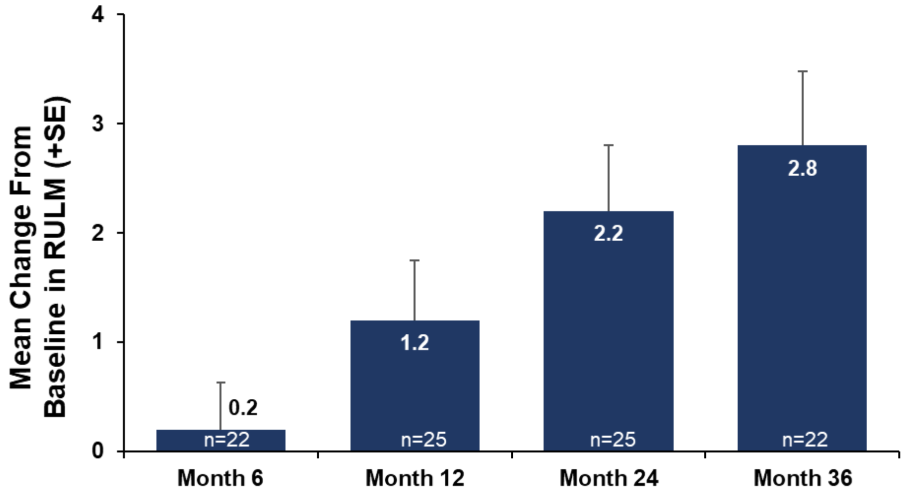

Error bars represent standard error of means. This analysis population included patients receiving either low-dose (2 mg/kg) or high-dose (20 mg/kg) apitegromab (inclusive of patients in Cohort 3 who transitioned from 2 mg/kg to 20 mg/kg in Year 2). This analysis excludes data post scoliosis surgery from 7 patients. RULM, Revised Upper Limb Module; SE, standard error; SMA, spinal muscular atrophy.
